# Supplementary material for: ACADL Promotes the Differentiation of Goat Intramuscular Adipocytes
Source: Animals (Basel). 2023 Jan 12;13(2):281. doi: 10.3390/ani13020281 (PMC9854987; doi:10.3390/ani13020281)
Supplement: Supplementary file 1 [file animals-13-00281-s001.zip › animals-2113303-supplementary.pdf]

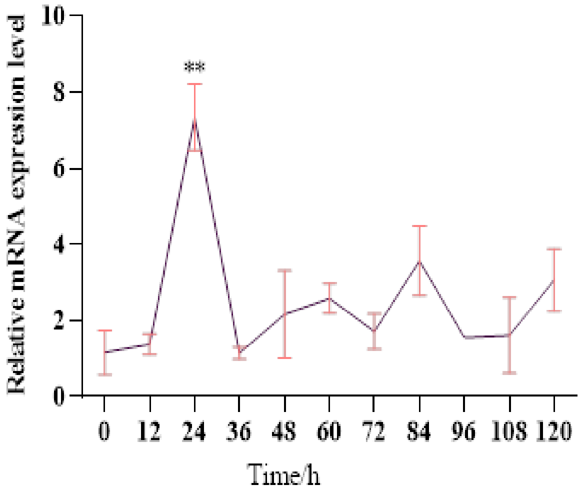

Figure S1. ACADL's expression level during intramuscular adipocyte differentiation.

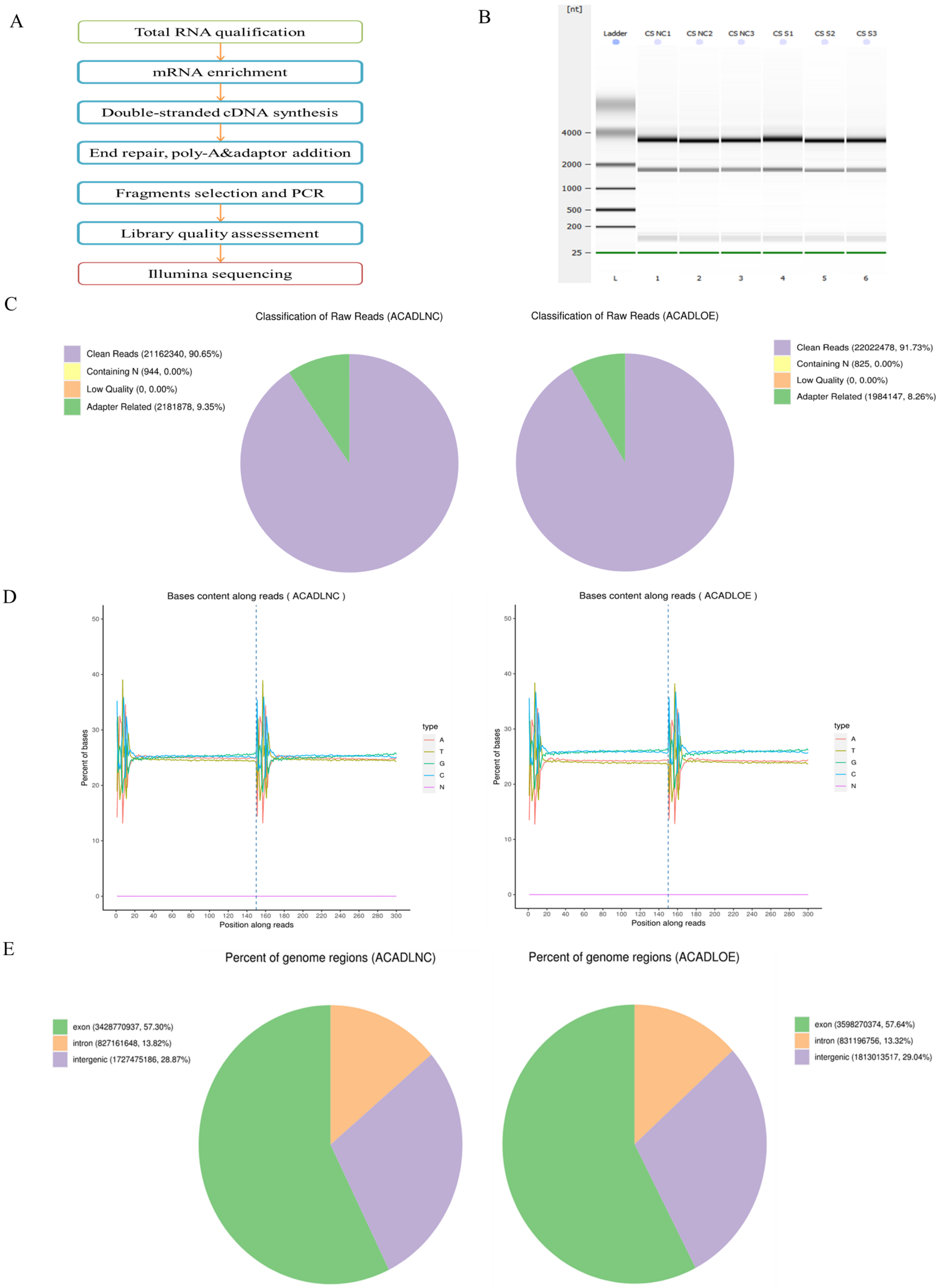

Figure S2. The quality control of RNA-sequencing A- Hands-on sequencing steps. B- Agarose gel electrophoresis plot. Ladder marker CS NC1-3: Control group (NC); CS S1-3: Test group (JN). C- Sequencing data's filtering. D- The content of GC. E-Sequencing reads in the genome region. exon: The number of reads compared to the exon region of the genome and its proportion of the number of clean reads. Intron: Compare the number of reads to the intron region of the genome and their proportion of clean reads. Intergenic: Compare the number of reads with cross- gene regions and its proportion of clean reads.

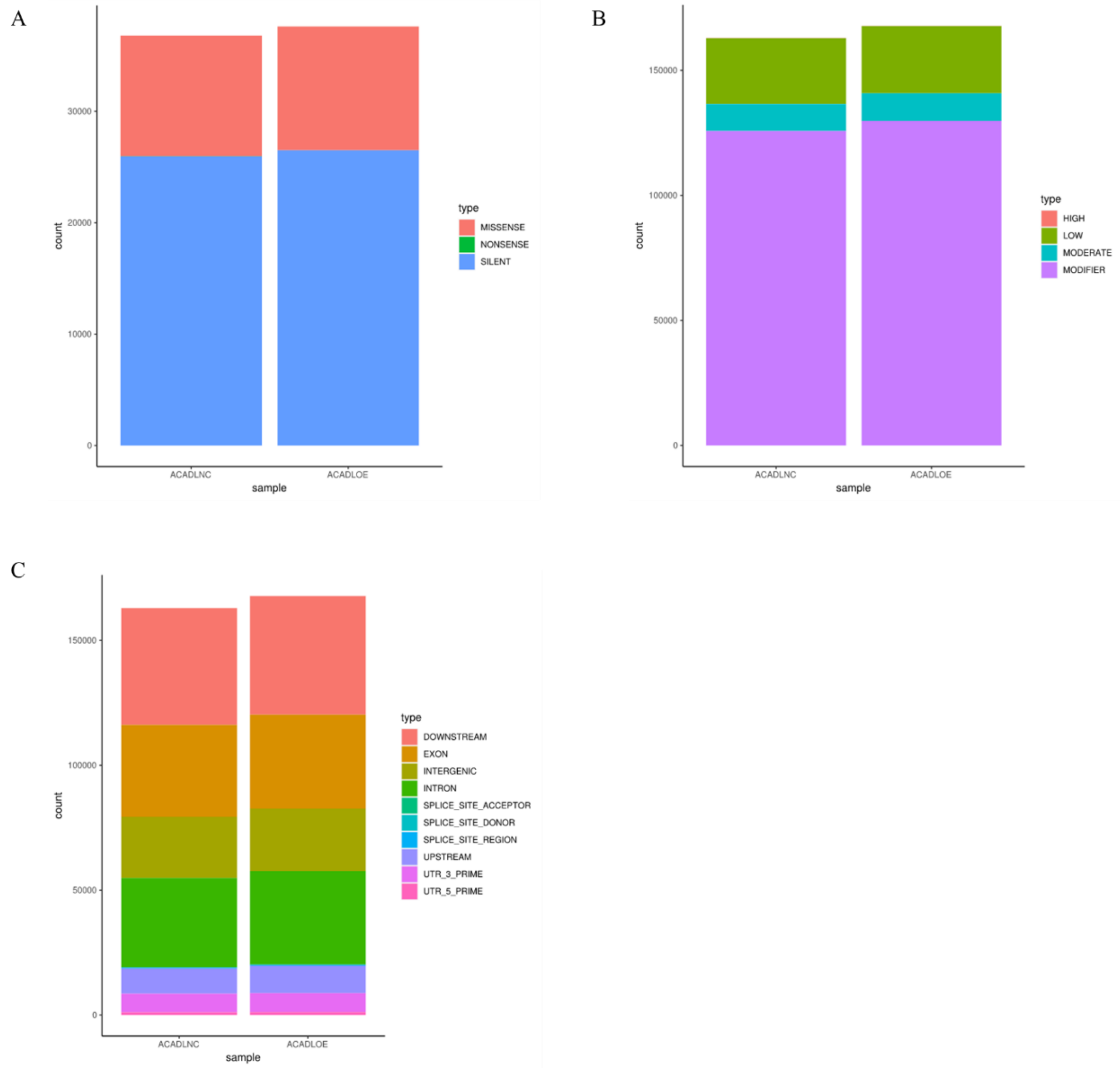

Figure S3 A-C.Single nucleotide polymorphism (SNP). Mutation site statistics. A- SNP impact. B-SNP function. C-SNP region.
